# A sub-chronic toxicity evaluation of a natural astaxanthin-rich carotenoid extract of *Paracoccus carotinifaciens* in rats

**DOI:** 10.1016/j.toxrep.2014.08.008

**Published:** 2014-08-25

**Authors:** Toyohisa Katsumata, Takashi Ishibashi, David Kyle

**Affiliations:** a Gotemba Laboratory, BoZo Research Center Inc., 1284 Kamado, Gotemba, Shizuoka 412-0039, Japan; b Biotechnology Business Group, Biotechnology Business Unit, Specialty Chemicals & Materials Company, JX Nippon Oil & Energy Corporation, 6-3, Otemachi 2-chome, Chiyoda-ku, Tokyo 100-8162, Japan; c Kyle Consulting Services, P. O. Box 119, Gualala, CA 95445, United States

**Keywords:** *Paracoccus carotinifaciens*, Sub-chronic toxicity, Astaxanthin, Rat

## Abstract

Astaxanthin is believed to be beneficial to human health because it possesses strong antioxidant properties. A natural astaxanthin-rich carotenoid extract (ARE) was produced by a well-controlled fermentation of a natural bacteria *Paracoccus carotinifaciens*, followed by the extraction and enrichment of the final product comprising mixture of carotenoids that is predominantly astaxanthin. The aim of this study was to evaluate the sub-chronic toxicity of the ARE using 6 week old Sprague-Dawley SPF rats [Crl:CD(SD)]. The test article was suspended in olive oil and administered daily to the rats by oral gavage for 13 weeks at doses of 0 (olive oil), 250, 500 or 1000 mg/kg/day. Each group consisted of 10 animals of each sex. No deaths occurred and no treatment-related changes were observed in the detailed clinical observations, manipulative tests, grip strength, motor activity, body weights, food consumption, ophthalmology, urinalysis, hematology, blood chemistry, organ weight, necropsy or histopathology. Dark-red feces were observed throughout the administration period in all treated groups due to excretion of the colored test article. Based on these results, it was concluded that the no observed adverse effect level (NOAEL) for ARE was at least 1000 mg/kg/day for male and female rats, respectively.

## 1 Introduction

Astaxanthin is an oxycarotenoid (*i.e*., possesses oxygen functional groups), and part of the xanthophyll group of carotenoids believed to provide health benefits by decreasing the risk of disease in humans [1], [2], [3], [4], [5]. Astaxanthin is also one of the dominant carotenoid contributors to the orange/red coloration found in salmon, shrimp and krill. The same coloration can be transferred to animals that eat large amounts of these astaxanthin-rich food sources (*e.g*., flamingoes). The ultimate producers of oxycarotenoids however are photosynthetic or microbial species that can be found in the aquatic environment (*e.g*., algae) or as terrestrial plants and microorganisms. Although we recognize the presence of astaxanthin by its red or orange color, its cellular biochemical role is thought to be based on the powerful antioxidant characteristics of this molecule [6], [7], [8], [9].

**Fig. 1:**
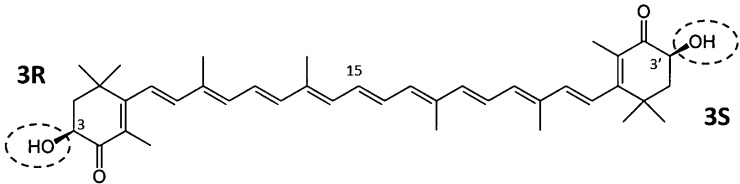
Chemical structure of astaxanthin showing the two chiral centers (3, 3′) in the 3R and 3′S form.

Astaxanthin can exist as several stereoisomers since the molecule has two chiral centers at carbons 3 and 3′ (Fig. 1). Both chiral centers can exist in either the *R* or *S* form and there are four possible stereo (or optical) isomers: *SS*, *RS*, *SR*, or *RR*. Astaxanthin can also be present in cells in a free form or esterified to certain fatty acids [10]. Astaxanthin produced by the algae *Heamatococcus pluvialis* is esterified and in the 3S, 3′S form, whereas that produced by the yeast *Phaffia rhodozyma* is nonesterified and in the 3R, 3R’ form, and that produced by the bacteria *Paracoccus carotinifaciens* is also nonesterified but in the 3S, 3′S form. Synthetic astaxanthin is used extensively worldwide as a coloring agent in salmon aquaculture and it is a racemic mixture of all optical isomers and in the non-esterified form. In wild Atlantic or Coho salmon the dominant form of astaxanthin is nonesterified and in the 3S, 3′S form similar to that of *P. carotinifaciens*. These nuances in the chemical structure of astaxanthin can, and has been used as a fingerprint to establish the source of pigmentation (synthetic or natural) in farm raised fish [11].

**Fig. 2:**
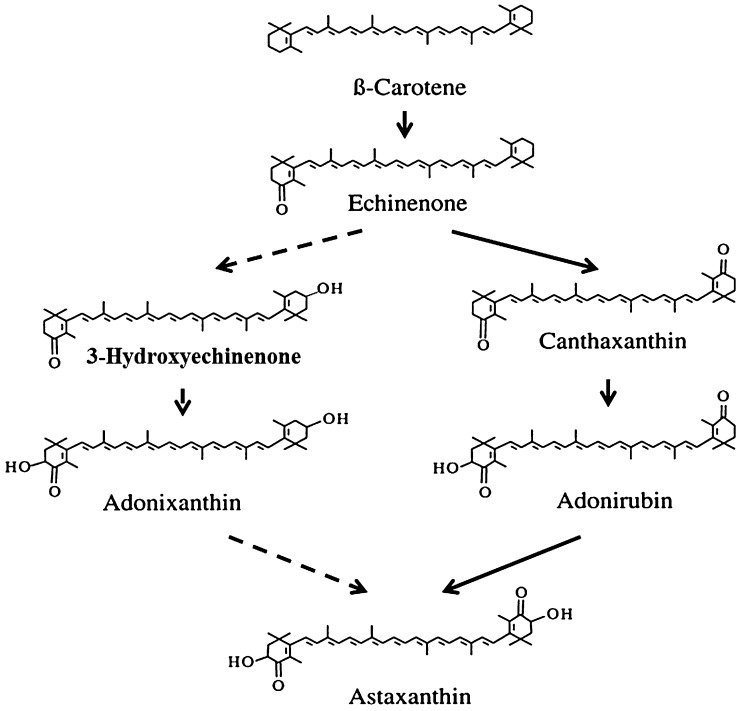
Proposed biosynthetic pathways from B-carotene to astaxanthin in *Paracoccus carotinifaciens* indicating the primary flux pathway (solid lines) and minor flux pathway (dashed line) and demonstrating the intermediate carotenoids present in the astaxanthin-rich extract test material.

*P. carotinifaciens* is a gram-negative, aerobic, rod-shaped bacterium with a high content of natural carotenoid. It is a member of the *alpha*-3 subclass of the *Proteobacteria* and classified in the genus *Paracoccus* based on the organism's genotypic and phenotypic characteristics and DNA composition [12]. Both the alga *H. pluvialis* (commercially sold as NaturRose^®^) and yeast *P. rhodozyma* (commercially sold as Ecotone^®^) contain astaxanthin as the vast majority of the total carotenoid in the cells (92% and 73% respectively), but the relatively low total carotenoid content of the cells (1.75% and 0.62% by weight respectively) results in a relatively low absolute Astaxanthin level on a gram dry weight basis (*i.e*., 1.6% and 0.45% respectively). The carotenoids present in *P. carotinifaciens* (commercially sold as Panaferd^®^) on the other hand, represent a range of natural carotenoids in the biosynthetic pathway leading to astaxanthin (Fig. 2), some of which are known to have a considerably higher antioxidant potential than astaxanthin itself [13]. Although the astaxanthin only represents 55–60% of the total carotenoid of the cells, the high total carotenoid level (4.1% by weight) results in a final astaxanthin level of 2.2% of the biomass.

Several toxicology studies including *in vitro* and *in vivo* mutagenicity studies, as well as repeat-dose toxicity studies in rats, using intact *P. carotinifaciens* dry cells have been performed to assess the safety of the whole cell biomass as a potential natural pigment source for the coloration of farm raised salmon and trout [14]. No notable toxicities for this organism have been observed and, as a result, the dry cell biomass has been approved for use as a natural coloring agent in salmon and trout aquaculture by the United States Food and Drug Administration (US FDA) as well as the European Food Safety Authority (EFSA). For the past 10 years, this whole cell, astaxanthin-rich material known commercially as Panaferd^®^ has been used extensively in the production of organic salmon and trout intended for human consumption.

With the growing interest in the antioxidant nature of astaxanthin [15], extracts from algal and yeast sources have been prepared and marketed worldwide as a dietary supplement for human use [16]. Such extracts are generally provided in an oleoresin format, but microencapsulated powders containing the extract are also available on the market. As an alternative to the algal and yeast sources, we have developed a process for the extraction and purification of the carotenoid pigments produced naturally in *P. carotinifaciens*. This extract has been shown to exert a protective effect (likely to its antioxidant capability) against induced ulcer formation in a murine gastric ulcer model [17]. As part of a pre-market safety assessment of this Astaxanthin-Rich carotenoid Extract (ARE) from *P. carotinifaciens*, a 13-week oral toxicity study was conducted in rats. This study was conducted in accordance with guidelines established by the Organization for Economic Co-operation and Development (OECD) including the Principle of Good Laboratory Practice (November 26, 1997) and Guidelines for the Testing of Chemicals 408 (September 21, 1998) relating to animal welfare. The study was undertaken under the approval of Institutional Animal Care and Use Committee (IACUC) of the IACUC of testing facility (Gotemba Laboratory, Japan). The ARE test material used in this study is a dark red powder which is extracted and purified from the whole cells of *P. carotinifaciens* and is more than 90% total carotenoids by weight comprising primarily astaxanthin, but also containing adonirubin, adonixanthin, canthaxantin, echinenone, 3-hydroxyechinenone, adonixanthin and beta-carotene.

## 2 Materials and methods

### 2.1 Preparation of the astaxanthin-rich extract from *P. carotinifaciens*

*P. carotinifaciens* biomass was produced by conventional stirred tank aerobic fermentation in a nutrient-rich liquid medium comprising glucose, ammonia, phosphate, minerals and vitamins [18]. The pH and concentration of dissolved oxygen was controlled to optimize the composition of carotenoids and the production of astaxanthin. At the end of the fermentation period, the mixture was heat-treated at 80 ± 5 °C for 30 ± 10 min to kill the *P. carotinifaciens* cells and inactivate endogenous enzymes. The liquid mixture of cells and fermentation broth was then filtered to remove much of the spent medium and washed with process water. The concentrated *P. carotinifaciens* cells were then dried and milled. The dried and milled cell powder was mixed to homogeneity (small, dark-red granules containing approximately 2% astaxanthin), weighed, and packaged in aluminum film bags.

The dried *P. carotinifaciens* whole cell powder was suspended in hot ethyl alcohol (95–100% in water) in an extraction tank to extract the carotenoids. The liquid mixture was then cooled and filtered to remove the residual biomass. The filtrate was concentrated and the carotenoids were allowed to crystallize. The pure crystallized carotenoid mixture was then collected by filtration and dried. The resulting ARE (the test material produced by the fermentation and extraction processes described above) used in this study (lot number CK-007) had a black-purple crystalline appearance. Chemical analysis of the test material indicated that it contained 904.8 mg/g of total carotenoid and 596.0 mg/g astaxanthin in addition to a number of other xanthophylls as shown in Table 1. The other components of the ARE included 8% lipid, 1% protein and about 1% ash. The phase transition temperature of the test material as measured by differential scanning calorimetry (DSC) was 222 °C, and the average particle size (D_50_) was 59 μm. The shelf life of the ARE at 25 °C in an unopened aluminum film bag was 24 months.

**Table 1:** Carotenoid structures and concentrations (% of dry weight) in the astaxanthin-rich extract test material from *Paracoccus carotinifaciens*.

Carotenoid	wt%	Structure
Astaxanthin	60	
Adonirubin	18	
Adonixanthin	5	
Canthaxanthin	5	
Echinenone	2	

### 2.2 Preparation of formulations containing the test material

The test formulations used for dosing were prepared by suspending the ARE test material in olive oil (Japanese Pharmacopoeia, Maruishi Pharmaceutical Co., Ltd.). For each dose concentration, a requisite amount of the test material was weighed, suspended in the olive oil and then diluted to a specified volume. Test suspensions were prepared once a week, transferred to brown glass bottles, and stored in a refrigerator (4–7 °C) until used. The test formulations were found to be perfectly stable under these conditions and each formulation was tested for concentration and homogeneity at each dose level in week 1 and week 13 of the study by high performance liquid chromatography (HPLC) using the following measuring conditions: column: Inertsil SIL 100A (4.6 mm i.d. × 250 mm, 5 μm, GL Sciences Inc., Japan) maintained at room temperature; Mobile phase: hexane/tetrahydrofuran/methanol (40:20:1); flow rate: 1.0 mL/min; detection: 470 nm; auto sampler temp: 5 °C; injection volume: 20 μL; measurement time: 18 min. The measured concentrations of the test suspensions were within a range of from 96.2 and 106.8% of the expected concentrations and with an acceptable coefficient of variation (CV) of from 0.4 to 1.5%.

### 2.3 Animals and husbandry

Five-week-old male and female specific pathogen free (SPF) Sprague-Dawley (SD) rats were purchased from Charles River Laboratories (Japan) and quarantined/acclimated for 9 days prior to the start of the study. The animals were housed in an animal room maintained at 22 ± 3 °C, with a relative humidity of 50 ± 20%, air ventilation at 12–17 vol/h, and with a 12-h light/dark cycle. All animals were housed individually in bracket-type stainless-steel wire mesh cages (W 254 × D 350 × H 170 mm: Lead Engineering Co., Ltd.) and allowed free access to a pelleted radiation-sterilized diet (CR-LPF, Oriental Yeast Co.) and to Gotemba City tap water *via* an automatic water supply system.

### 2.4 Experimental procedure

The test material dosing volume was 10 mL/kg body weight for all animals using the ARE dose levels set at 0 (olive oil), 250, 500 and 1000 mg/kg/day. The test material was administered on a daily basis by oral gavage using a stomach tube for a period of 13-weeks. All animals were observed 3 times a day during the administration period (prior to, immediately after, and approximately 1–3 h after dosing). On the days of the detailed clinical observation the animals were only observed twice a day (prior to, and immediately after dosing). Detailed clinical observations were conducted once before the start of administration, so that any animals that showed abnormalities could be excluded from the study, and once weekly after the daily dosing during the administration period. Detailed clinical observations consisted of a home cage observation (posture, convulsion, abnormal behavior), an in-the-hand observation (ease of removal from cage, condition of fur and skin, secretions from eyes and nose, exophthalmos, palpebral closure, visible mucous membrane, autonomic nervous function (lacrimation, salivation, piloerection, pupil size and abnormal respiration), reactivity to handling, and vocalization at handling), and an open field observation (arousal, convulsion, abnormal behavior, stereotypy, gait, posture, grooming, rearing count and excretion (defecation count and urination)). In the manipulative test, measurements of grip strength and motor activity were conducted in week 12 of the study. The manipulative test consisted of auditory responsiveness, approach responsiveness, touch responsiveness, pain responsiveness, pupillary reflex, aerial righting reflex, and landing foot splay. Grip strength of forelimbs and hind limbs was measured by a digital push pull strain gauge (MODEL-RX-5; AIKOH Engineering Co., Ltd., Japan). Locomotor activity was measured for 1 h using an infrared beam sensor for experimental animals (NS-AS01 from NeuroScience, Inc, Japan), and values in 10-min intervals from 0 to 60 min were collected. Body weights were recorded every 3 or 4 days. Food consumption was recorded every 7 days. Water intake was measured at the time of urinalysis conducted in week 13 of the study.

An ophthalmological examination was conducted during the acclimation period (before the start of the administration period) and in week 13 of the study. Any animals with abnormalities identified in the acclimation period examination that could affect the toxicological evaluation were excluded from the study. All the males and females in the control group and the high dose group underwent the ophthalmological examination in week 13 immediately after the routine daily dosing. A mydriatic agent, Mydrin P (Santen Pharmaceutical Co., Ltd., Japan) was initially applied to the eyes for mydriasis and the anterior part of the eyes, optic media and fundus oculi of all animals were then examined using an ophthalmoscope (Omega 200: HEINE Optotechnik GmbH & Co., Germany).

Urinalysis was conducted in week 13 of the study. After dosing on the day of the examination, all animals were individually placed in cages equipped with a urine collector under deprivation of food but with free access to water, and 4-h urine samples were collected. A 20-h urine sample was also collected after free access to food and water. Examination of pH, protein, ketones, glucose, occult blood, bilirubin and urobilinogen was done on the first 4-h urine samples using a urine chemistry autoanalyzer (CLINITEK 500, Siemens Healthcare Diagnostics Inc., USA). Color was examined macroscopically, and urinary sediments were examined microscopically on the first 4-h urine samples. Osmotic pressure was measured on the 20-h urine samples using an automatic osmotic pressure meter (OSMO STATION OM-6060, Arkray Inc., Japan). The electrolytes (sodium, potassium and chloride) were determined on the 20-h urine samples using a clinical chemistry autoanalyzer (TBA-120FR, Toshiba Medical Systems Corporation, Japan). One day's urine volume was calculated by totaling the 4-h urine and 20-h urine samples. The daily excretion of electrolytes was calculated directly from the determined concentration and urine volume over this 24 h period.

The hematological sampling and assessment was done at necropsy on the day following the end of the administration period. All animals were deprived of food overnight (approximately 16 h) and subjected to abdominal incision under isoflurane anesthesia. Blood samples were collected from the abdominal aorta into blood collection bottles containing an anticoagulant (EDTA-2K) and analyzed using an Adiva 120 Hematology System (Siemens Healthcare Diagnostics Inc., USA) for red blood cell count (RBC), hemoglobin (HGB), hematocrit (HCT), mean corpuscular volume (MCV), mean corpuscular hemoglobin (MCH), mean corpuscular hemoglobin concentration (MCHC), reticulocyte percentage (Retic), platelet count (PLT), white blood cell count (WBC), and differential white blood cell percentage and count. Further, blood samples were collected into tubes containing 3.8% sodium citrate, and plasma obtained by centrifugation was examined using a coagulometer (ACL Elite Pro, Instrumentation Laboratory, UK) for prothrombin time (PT), activated partial thromboplastin time (APTT) and fibrinogen (FIB).

For blood chemistry, blood samples were collected from the abdominal aorta into blood collection bottles containing an anticoagulant (heparin sodium) and analyzed using a clinical chemistry autoanalyzer (TBA-120FR, Toshiba Medical Systems Corporation, Japan) for aspartate aminotransferase (AST), alanine aminotransferase (ALT), lactate dehydrogenase (LDH), alkaline phosphatase (ALP), total cholesterol (T-CHO), triglyceride (TG), phospholipids (PL), total bilirubin (T-BIL), glucose (GLU), blood urea nitrogen (BUN), creatinine (CRNN), sodium (Na), potassium (K), chloride (Cl), calcium (Ca), inorganic phosphorus (P), total protein (TP), albumin (ALB) and A/G ratio (A/G).

Gross pathology was examined at necropsy and the organs were weighed (absolute weight) and fixed in 10% formalin (vol/vol), and the weight per 100 g body weight (relative weight) was calculated based on the fasted animal's body weight and absolute organ weight. The gross pathology of the brain (cerebrum + cerebellum), adrenals, thymus, spleen, heart, liver, kidneys, testes, epididymides, ovaries and uterus was observed and measured. A number of organs and tissues were also fixed in 10% formalin (vol/vol). These included medullary, pons, spinal cord (cervical, midthoracic and lumbar), sciatic nerves, Harderian glands, pituitary, thyroids, parathyroids, submandibular lymph node, mesenteric lymph node, thoracic aorta, trachea, lung (including bronchus), tongue, esophagus, stomach, duodenum, jejunum, ileum (including Peyer's patch), cecum, colon, rectum, submandibular glands, sublingual glands, pancreas, urinary bladder, prostate, seminal vesicles, vagina, mammary glands (inguinal region), sternum (including bone marrow), femurs (including bone marrow), femoral skeletal muscles and skin (inguinal region). The eyeballs and optic nerves were fixed in glutaraldehyde/formalin (3%/2% vol/vol in water) and the testes and epididymides in Bouin's solution first, and then preserved in phosphate buffered 10% formalin (vol/vol). All preserved organs and tissues in the control and high dose groups (1000 mg/kg) were sectioned and stained with hematoxylin and eosin (H&E) and examined for histopathology.

### 2.5 Statistical analysis

Numerical data obtained for respective parameters (quantitative items of open field observations, quantitative items of manipulative tests, measurements of grip strength and motor activity, body weight, food consumption, water intake and quantitative items of urinalysis as well as data from hematology and blood chemistry and organ weight) were analyzed using the Bartlett test, Dunnett test, or Steel test [19].

## 3 Results

No deaths occurred in any of the groups throughout the administration period. Excretion of dark-red feces was observed throughout the administration period in all males and females in all treated groups. In the detailed clinical observations, no treatment-related changes were observed. In the open field observations, a low value in the rearing count was recorded in week 3 of administration in females in the mid-dose group (500 mg/kg) with statistical significance. However, it was judged to be incidental since it was not dose-related. In the manipulative tests, measurements of grip strength, and motor activity, no test material related changes were observed.

**Fig. 3:**
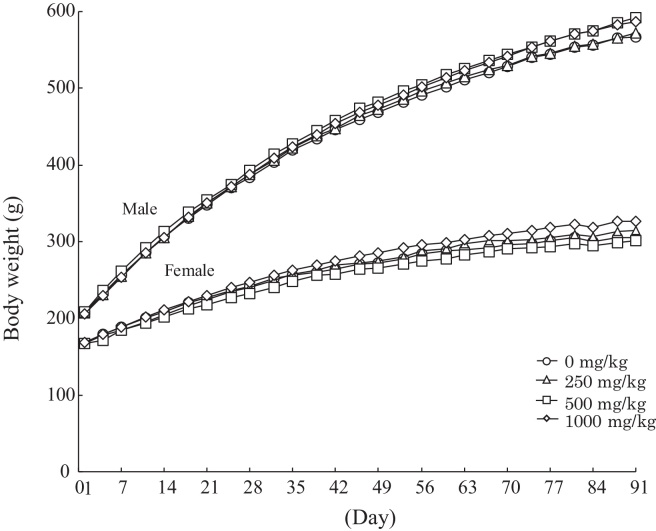
Animal growth curves for a 13-week repeated oral dose toxicity study of a natural astaxanthin-rich carotenoid extract in rats.

**Fig. 4:**
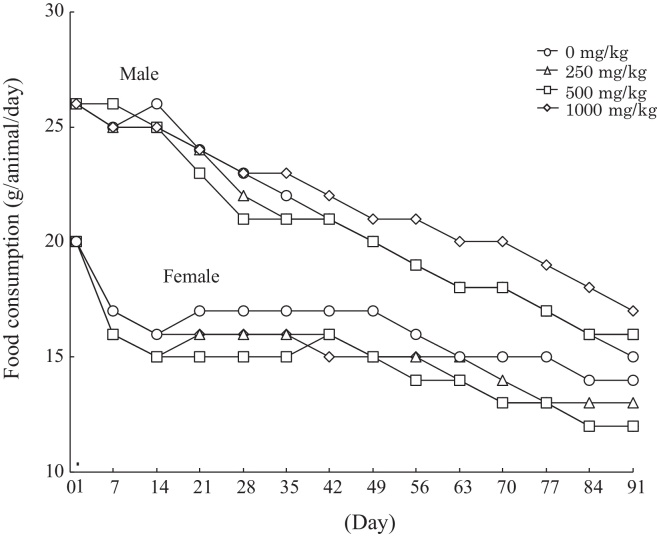
Food consumption in animals during a13-week repeated oral dose toxicity study of a natural astaxanthin-rich carotenoid extract in rats.

There were no treatment-related changes in body weight gain in any of the groups and the growth curves of the male and female animals during the study were consistent with historical background data from this test facility (Fig. 3). The food consumption data (Fig. 4) also tracked historical norms from this facility, but statistical analysis revealed that higher food consumption was observed in the high dose (1000 mg/kg) male group on day 84 and in the high dose (1000 mg/kg) female group on day 77. However, these changes were judged to be incidental fluctuations since they were observed at only one time point during the study. Likewise a low food consumption recorded on day 14 of administration in females in the 500 mg/kg group was also judged to be incidental since it was a single point and not dose-related.

No treatment-related changes were observed in ophthalmology, urinalysis (including water intake), or hematology in males or females in any treatment group. However, male animals in each dose group showed significant prolongation or tendency toward prolongation of prothrombin time, but this did not seem to be dose dependent on the test material nor was it apparent in the female animals (Table 2). Male animals in the high dose group (1000 mg/kg) had a statistically significant prolongation of activated partial thromboplastin time. These values, however, were within the normal range of the historical background data of the test facility. Male animals in the mid-dose group (500 mg/kg) showed a significantly higher value in the percentage of eosinophils in leukocyte percentage, and females in the low dose group (250 mg/kg) showed a significantly lower value in fibrinogen. Both of these observations were unrelated to the dose of the test material.

**Table 2:** Hematology parameters at the conclusion of a 13-week repeated oral dose toxicity study of a natural astaxanthin-rich carotenoid extract in rats.

Sex	Dose (mg/kg)	No. of rats examined	Eos ratio (%)	PT (s)	APTT (s)	FIB (mg/dL)
Male	0	10	1.1 ± 0.3	12.0 ± 0.8	17.2 ± 2.2	271 ± 22
	250	10	1.1 ± 0.4	13.4 ± 0.9**	18.5 ± 1.9	269 ± 27
	500	10	1.5 ± 0.3*	14.1 ± 2.2**	19.5 ± 1.8	262 ± 26
	1000	10	1.3 ± 0.5	13.7 ± 1.9	19.8 ± 2.5*	272 ± 21

Female	0	10	1.3 ± 0.4	11.1 ± 0.6	14.2 ± 1.4	177 ± 14
	250	10	1.4 ± 0.3	10.9 ± 0.6	13.7 ± 1.5	160 ± 14*
	500	10	1.6 ± 0.5	10.8 ± 0.4	14.5 ± 1.5	170 ± 15
	1000	10	1.6 ± 0.3	10.9 ± 0.6	13.7 ± 1.4	182 ± 18

* *p* ≤ 0.05 (significantly different from the control group mean).

** *p* ≤ 0.01 (significantly different from the control group mean).

There were no treatment-related changes in blood chemistries, gross pathology or histopathology of in any of the animals in any of the study groups. Likewise, no treatment-related changes were observed in organ weights. Although a statistically significant higher value in the absolute weight of the brain was recorded in males in the low dose group (250 mg/kg) and a low value in the relative spleen weight in males in the mid dose group (500 mg/kg), they were considered to be incidental changes only in the absolute or relative weight and were not related to the dose of the test material.

## 4 Discussion

In this study, the only treatment-related changes observed were the excretion of dark-red feces in all males and females in all treated groups throughout the administration period. However, this was due to excretion of the colored test material into feces and was not suggestive of toxicity. Based on these results, the 13-week repeated dose oral administration of a natural astaxanthin-rich extract (ARE) from *P. carotinifaciens* to rats at dose levels of 250, 500 and 1000 mg/kg/day was not associated with any significant toxic changes. Therefore, it was concluded that the no observed adverse effect level (NOAEL) in this study was at least 1000 mg/kg/day for both males and females.

These data are consistent with previous observations and conclusions for the whole *P. carotinifaciens* cell material now used extensively in the organic aquaculture industry [14]. They are also consistent with studies on other astaxanthin-containing microbes such as *H. pluvialis* [20] and *P. rhodozyma* [21] when the whole biomass itself has been tested. Although the oleoresin extracts from algal and yeast sources are presently on the market, we are unaware of any previously published subchronic toxicological evaluation in rats similar to the present study that has been completed for those sources of astaxanthin. Nevertheless, the present study does demonstrate that with the purified astaxanthin-rich extract from *P. carotinifaciens*, there appear to be no significant observable adverse effects in rats when consumed at dose levels considerably higher than levels being used presently by the supplements industry (5–15 mg/person/day).

## Conflict of interest

Dr. Katsumata is paid employee at the BoZo Research Center who conducted the work under contract to JX-NOE. Dr. Ishibashi is an employee of JX-NOE. Dr. David Kyle acts as a paid consultant for JX-NOE.

## Transparency document

Transparency document
